# Statins and the progression of age-related macular degeneration in the United States

**DOI:** 10.1371/journal.pone.0252878

**Published:** 2021-08-04

**Authors:** Cassie A. Ludwig, Daniel Vail, Nitya A. Rajeshuni, Ahmad Al-Moujahed, Tatiana Rosenblatt, Natalia F. Callaway, Malini Veerappan Pasricha, Marco H. Ji, Darius M. Moshfeghi

**Affiliations:** 1 Department of Ophthalmology, Byers Eye Institute, Stanford University, Palo Alto, CA, United States of America; 2 Department of Ophthalmology, Retina Service, Massachusetts Eye and Ear, Harvard Medical School, Boston, MA, United States of America; Medical Research Foundation (Sankara Nethralaya), INDIA

## Abstract

**Purpose:**

To study the effect of statin exposure on the progression from non-exudative to exudative age-related macular degeneration (AMD).

**Methods:**

Retrospective cohort study of commercially insured patients diagnosed with non-exudative AMD (n = 231,888) from 2007 to 2015. Time-to-event analysis of the association between exposure to lipid-lowering medications and time from non-exudative AMD to exudative AMD diagnosis was conducted. Outcome measures included progression to exudative AMD, indicated by diagnosis codes for exudative AMD or procedural codes for intravitreal injections.

**Results:**

In the year before and after first AMD diagnosis, 11,330 patients were continuously prescribed lipid-lowering medications and 31,627 patients did not take any lipid-lowering medication. Of those taking statins, 21 (1.6%) patients were on very-high-dose lipophilic statins, 644 (47.6%) on high-dose lipophilic statins, and 689 (50.9%) on low-dose lipophilic statins. We found no statistically significant relationship between exposure to low (HR 0.89, 95% CI 0.83 to 1.38) or high-dose lipophilic statins (HR 1.12, 95% CI 0.86 to 1.45) and progression to exudative AMD. No patients taking very-high-dose lipophilic statins converted from non-exudative to exudative AMD, though this difference was not statistically significant due to the subgroup size (p = .23, *log-rank test*).

**Conclusions:**

No statistically significant relationship was found between statin exposure and risk of AMD progression. Interestingly, no patients taking very-high-dose lipophilic statins progressed to exudative AMD, a finding that warrants further exploration.

## Introduction

Age-related macular degeneration (AMD) is a neurodegenerative disease of the macula in which lipid-rich protein deposits accumulate between the retinal pigmented epithelium (RPE) and Bruch’s membrane (BrM). These lipid deposits interact with reactive oxygen species to form peroxidized lipids that either trigger the inflammatory pathway through upregulation of both cytokines and vascular endothelial growth factor (VEGF), which may lead to choroidal neovascularization, or cause toxicity to RPE cells leading to geographic atrophy [1–3].

While AMD ultimately results in neurodegeneration, AMD likely begins in the choroidal vasculature. The vascular model of macular degeneration posits that impairment of choroidal perfusion of the RPE leads to accumulation of lipoproteins in drusen and BrM [4]. Prior to the development of drusen, cholesterol accumulates in the same location suggesting its role in the pathogenesis of drusen and ultimately AMD [5]. AMD bears many similar risk factors to cardiovascular disease including age, smoking, obesity, hypertension, and atherosclerosis [6]. Since statins are a first-line therapy for hypercholesterolemia and coronary artery disease, statins may also aid in preventing or halting AMD pathogenesis [7].

As 3-hydroxy-3-methylglutaryl-coenzyme A (HMG-CoA) reductase inhibitors, the primary function of statins is to lower lipid levels by inhibiting the conversion of HMG-CoA into L-mevalonic acid, thereby inhibiting the production of isoprenoid intermediates of the biosynthetic cholesterol pathway [8]. Secondarily, statins have pleiotropic anti-inflammatory, anti-angiogenic, anti-thrombotic and antioxidant effects [9–13]. At present, the Age-Related Eye Disease Study (AREDS) vitamin formulation is the only intervention shown to prevent progression from non-exudative to exudative AMD and no treatment exists for geographic atrophy [14]. Given statins’ immunomodulatory and lipid-lowering effects, prior studies have examined their potential as therapy for non-exudative AMD with contradictory results [15–23]. These studies, however, have been limited by small numbers, a lack of stratified analysis of low- and high-dose statins, or limited or no control for confounders. We hypothesized that, after appropriate control for confounders available in the present database, patients with non-exudative AMD on very-high-dose lipophilic statins would have reduced progression to exudative AMD.

## Materials and methods

All data were fully anonymized before they were accessed for analysis. Institutional Review Board (IRB)/Ethics Committee ruled that approval was not required for this study.

### Study population

Patients were selected from the IBM MarketScan database, which includes administrative insurance data and prescription drug claims from 350 payers. Of the 123,637,719 commercially insured patients with outpatient encounters, 231,888 incident cases of non-exudative AMD occurred between 2008 and 2015. International Classification of Diseases (ICD) diagnostic codes versions 9 and 10 were used to identify diagnoses and FDA National Drug Codes (NDC) were used to identify prescriptions received and filled. Patients were included if they had a non-exudative AMD diagnosis (362.51, H35.31) and unchanged use of lipid-lowering medications (drug and dose) within a year of their index diagnosis. Early, intermediate, and advanced AMD stages were not distinguished, as the number of patients with ICD-10 coding was limited to 2015 to 2017. Exclusion criteria included patients with diabetes mellitus (250.00–250.93, E08-E13, O240-249), non-continuous database enrollment, < 365 days of coverage preceding or following index non-exudative AMD diagnosis, lack of medication data, or evidence of exudative AMD diagnosis or related treatment (procedure code for intravitreal injections) prior to diagnosis of non-exudative AMD. Patients were included in the control group if they had not taken a lipid-lowering medication within the year preceding or following their index non-exudative AMD diagnosis. Patients were included in the exposure group if they had a prescription for a lipid-lowering medication in both the year preceding and following their index diagnosis. Control group patients who began taking a lipid-lowering medication more than a year after their index diagnosis were censored from survival models at the date they began the medication. Similar methodology has previously been used to investigate the association between lipid-lowering medications and risk of diabetic retinopathy [24].

### Outcomes

The primary outcome was progression to exudative AMD, as indicated by diagnosis codes for exudative AMD or procedural codes for intravitreal injections. The earliest diagnosis or procedural code indicating exudative AMD was used to measure time from non-exudative AMD diagnosis to exudative AMD diagnosis.

### Exposures

To evaluate exposure to lipid-lowering medication prior to non-exudative AMD diagnosis, we coded individual medications using NDC numbers included in pharmaceutical records for each patient. Exposure was categorized based on use of any lipid-lowering medication, use of a statin, use of fibrates (fenofibrate, gemfibrozil), or concomitant use of both statins and fibrates. Statin exposure was further divided into hydrophilic statins (rosuvastatin, pravastatin) versus lipophilic statins (simvastatin, lovastatin, atorvastatin, fluvastatin, pitavastatin) [25, 26], with the latter category characterized based on dosage: 1) very-high-dose lipophilic statins (atorvastatin 80 mg) [23], 2) high-dose lipophilic statins (atorvastatin 20–40 mg, simvastatin 40–80 mg, pitavastatin 2–4 mg) [17], 3) low-dose lipophilic statins (atorvastatin 10 mg, simvastatin 5–20 mg, fluvastatin 20-80mg, lovastatin 10–80 mg, pitavastatin 1 mg) [27].

### Covariates

We adjusted for comorbidities associated with exudative AMD risk and with use of a lipid-lowering medication, including age at index non-exudative AMD diagnosis, sex, smoking, and a vector of comorbidities drawn from the Elixhauser Comorbidity Index [28]. Additionally, we adjusted for baseline use of certain medications prior to index diagnosis that indicated a more complicated cardiovascular history (anticoagulants, antihypertensive medications, or diuretics).

### Statistical analysis

All data were analyzed using Stata 14.2 (StataCorp, College Station, TX, US). We conducted time-to-event analysis of the association between exposure to lipid-lowering medications and time from diagnosis of non-exudative AMD to diagnosis of exudative AMD. We estimated Cox proportional hazards models for time to first diagnosis of exudative AMD and time to first intravitreal injection, including unadjusted and adjusted models using the aforementioned covariates. We also performed a sensitivity analysis using Cox proportional hazards models with propensity score matching and restriction of the sample to the region of common support (with inverse probability weighting).

## Results

We identified 231,888 patients with a diagnosis of non-exudative AMD, of whom 42,957 met criteria for analysis (**S1 Fig**). Demographic data is included in **Table 1**. Patients were started on lipid-lowering medications at an average age of 47 years (SD 2.1 years). Patients taking lipid-lowering medications were less likely to be female (51.2%) than those not taking lipid-lowering medications (63.6%, p < .001) and were more likely to be current or former smokers (5.9% vs. 4.5%, p < .001), had a higher average Elixhauser comorbidity index (2.2 vs. 1.5, p < .001), and were more likely to be taking an anticoagulant (3.2% vs. 1.6%, p < .001), antihypertensive (49.1% vs. 19.2%, p < .001), or diuretic (17.7% vs. 8.5%, p < .001).

**Table 1:** Characteristics: Baseline characteristics of patients diagnosed with non-exudative age-related macular degeneration between 2008 to 2015.

Characteristics	No lipid-lowering medication	Lipid-lowering medication	P Value
	(N = 31,627)	(N = 11,330)	
Age at diagnosis (mean, SD)	46.6 (2.1)	46.4 (2.1)	< .001
Female	20,125 (63.6)	5,800 (51.2)	< .001
Smoker	1,408 (4.5)	672 (5.9)	< .001
Years follow-up (mean, SD)	3.1 (1.7)	2.9 (1.6)	< .001
Total Elixhauser comorbidities (mean, SD)	1.5 (1.6)	2.2 (1.8)	< .001
On anticoagulation	512 (1.6)	366 (3.2)	< .001
On antihypertensive medication	6,085 (19.2)	5,566 (49.1)	< .001
On diuretics	2,702 (8.5)	2,001 (17.7)	< .001
**Exposure to Lipid-Lowering Medications**			
Any lipid-lowering medication		11,330 (100)	
Statin		9,211 (81.3)	
Fibrates		453 (4.0)	
Simultaneous statin and fibrates		192 (1.7)	
**Exposure to Statins by Lipophilic-Hydrophilic Balance**			
Lipophilic statins		6,731 (59.4)	
Hydrophilic statins		2,353 (20.8)	
**Exposure to Statins by Class**			
Very high dose lipophilic statin		21 (1.6)^a^	
High dose lipophilic statin		644 (47.6)	
Low dose lipophilic statin		689 (50.9)	
**Exposure to Statins by Drug**^**b**^			
Simvastatin (lipophilic)		3,725 (41.7)^c^	
Lovastatin (lipophilic)		318 (3.6)	
Atorvastatin (lipophilic)		2,515 (28.2)	
Fluvastatin (lipophilic)		24 (0.3)	
Pitavastatin (lipophilic)		16 (0.2)	
Rosuvastatin (hydrophilic)		1,305 (14.6)	
Pravastatin (hydrophilic)		1,026 (11.5)	
**Outcomes**			
Exudative macular degeneration	1,012 (3.2)	394 (3.5)	0.154
Anti-VEGF injection	2,004 (6.3)	777 (6.9)	0.053

SD = standard deviation; VEGF = vascular endothelial growth factor.

^a^Percentage of all patients on statins with NDC coding including dosing.

^b^In order from most to least lipophilic.

^c^Percentage of all patients on non-combination statins.

Of the 11,330 patients taking a lipid-lowering medication, 9,211 (81.3%) were taking a statin, 453 (4.0%) a fibrate, and 192 (1.7%) both a statin and fibrate. Of those taking statins, NDC coding included dosing for 1,354 patients (14.7%): 21 (1.6%) took very-high-dose lipophilic statins, 644 (47.6%) took high-dose statins, and 689 (50.9%) took low-dose statins. Most patients were prescribed lipophilic statins, with 3,725 of 8,929 patients (41.7%) on simvastatin, 2,515 (28.2%) on atorvastatin, 318 (3.6%) on lovastatin, 24 (0.3%) on fluvastatin, and 16 (0.2%) on pitavastatin. Of those taking a hydrophilic statin, the majority took rosuvastatin (n = 1,305, 14.6%) compared to pravastatin (n = 1,026, 11.5%).

### Progression from non-exudative to exudative macular degeneration

Overall, 8.3% (n = 945) of patients on lipid-lowering medications had a diagnosis code for exudative AMD or a procedural code for anti-VEGF injections following non-exudative AMD diagnosis, compared to 7.9% (n = 2,483) of patients who did not take lipid-lowering medications (p = .099). Average follow-up time was 1,066 days (SD 590 days) for those on lipid-lowering medications compared to 1,130 days (SD 632 days) for those not on lipid-lowering medications. In total, 3,079 patients were censored from our analysis as they eventually began taking a lipid-lowering medication. Among the control group, average time from diagnosis of non-exudative AMD to first lipid-lowering prescription was 930 days (SD 518 days).

**Fig 1:**
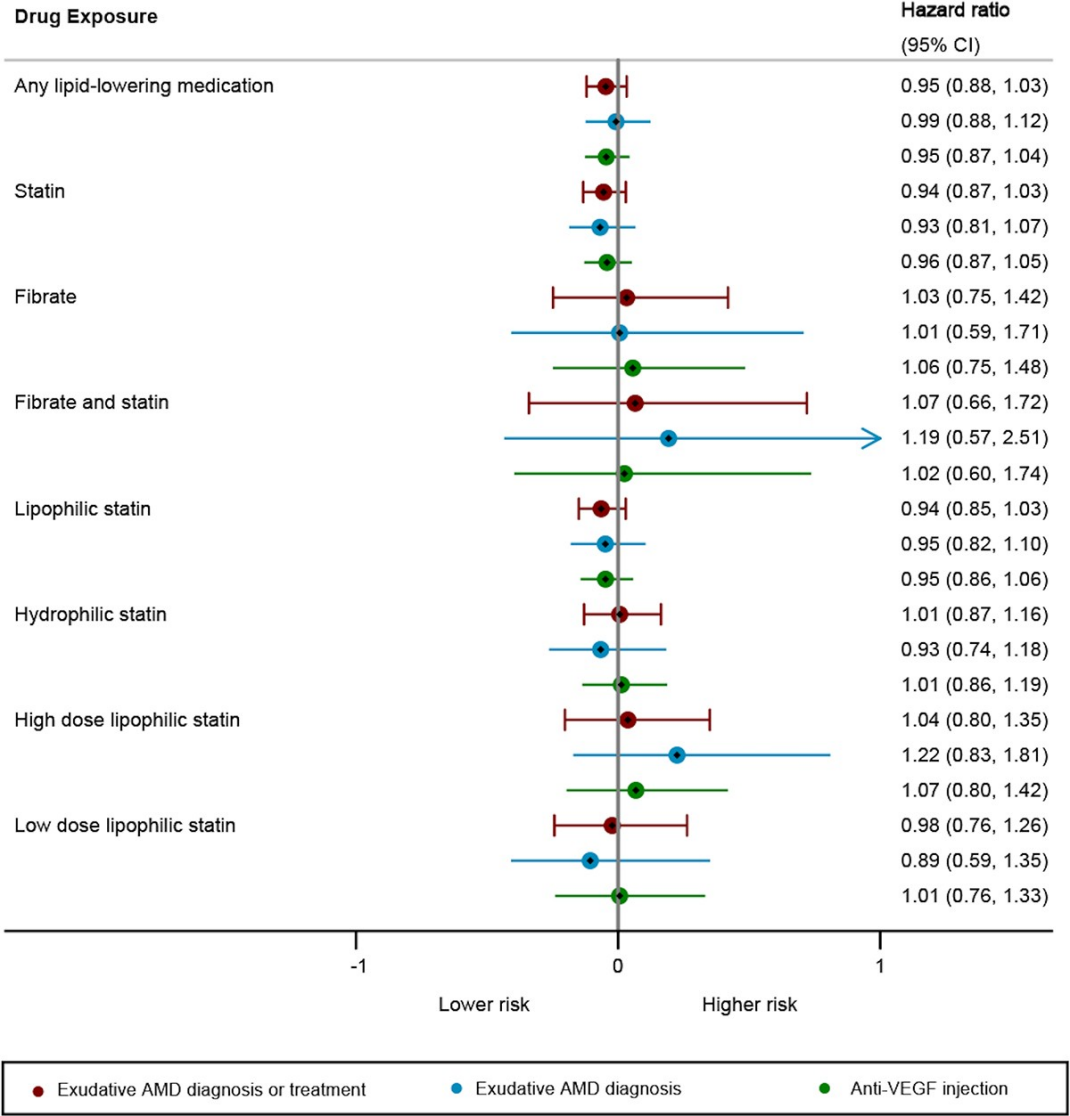
Forest plot demonstrating adjusted cox proportional hazards models of patients exposed to lipid-lowering medications and their progression to exudative AMD and/or need for treatment with anti-VEGF medication.

We found no significant difference in progression to exudative AMD or risk of receiving an anti-VEGF injection in patients who took lipid-lowering medications prior to diagnosis after adjusting for age at index diagnosis of non-exudative AMD, sex, smoking, comorbidities from the Elixhauser Comorbidity Index, and use of anticoagulants, antihypertensives, or diuretics (**Fig 1**). Similarly, no significant differences were found in progression to exudative AMD in those taking lipophilic statins (HR 1.03, 95% CI 0.90–1.19) or hydrophilic statins (HR 0.98, 95% CI 0.77–1.23).

We found no statistically significant subgroup, though no patients taking very-high-dose statins converted to exudative AMD (not shown here, as hazard ratios could not be calculated).

None of the patients taking very-high-dose lipophilic statins prior to non-exudative AMD diagnosis progressed to exudative AMD or required an anti-VEGF injection (p = .23, *log-rank test*). We found no statistical difference in risk of exudative AMD in those taking high-dose lipophilic statins (HR 1.12, 95% CI 0.86 to 1.45), low-dose lipophilic statins (HR 1.07, 95% CI 0.83 to 1.38), fibrates alone (HR 1.13, 95% CI 0.83 to 1.55), or both statins and fibrates (HR 1.18, 95% CI 0.73 to 1.91).

**Fig 2:**
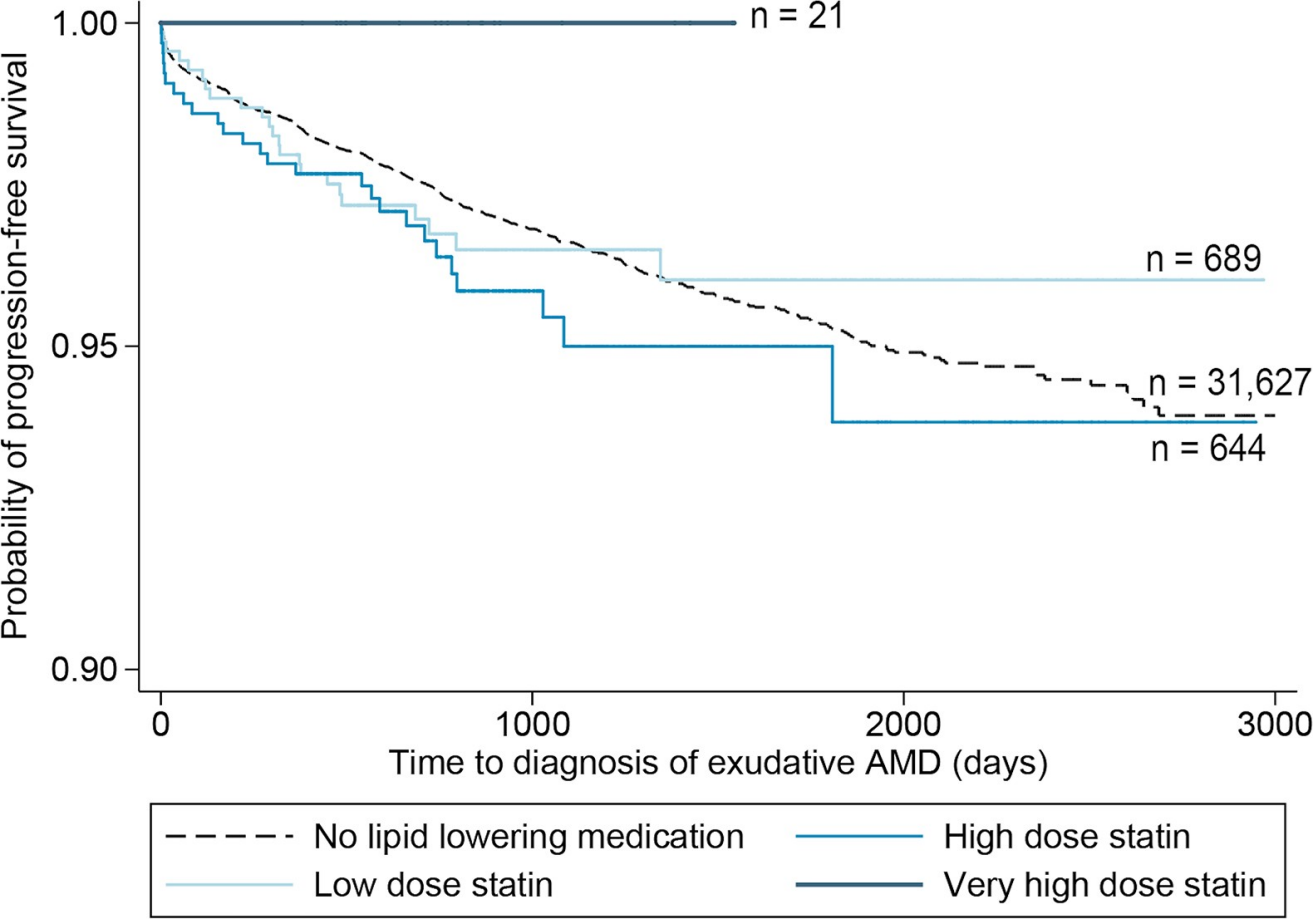
Time to progression from non-exudative to exudative age-related macular degeneration (AMD) by dose of lipophilic statin. No patients taking very-high-dose lipophilic statins (atorvastatin 80 mg) converted from non-exudative to exudative AMD.

**Fig 3:**
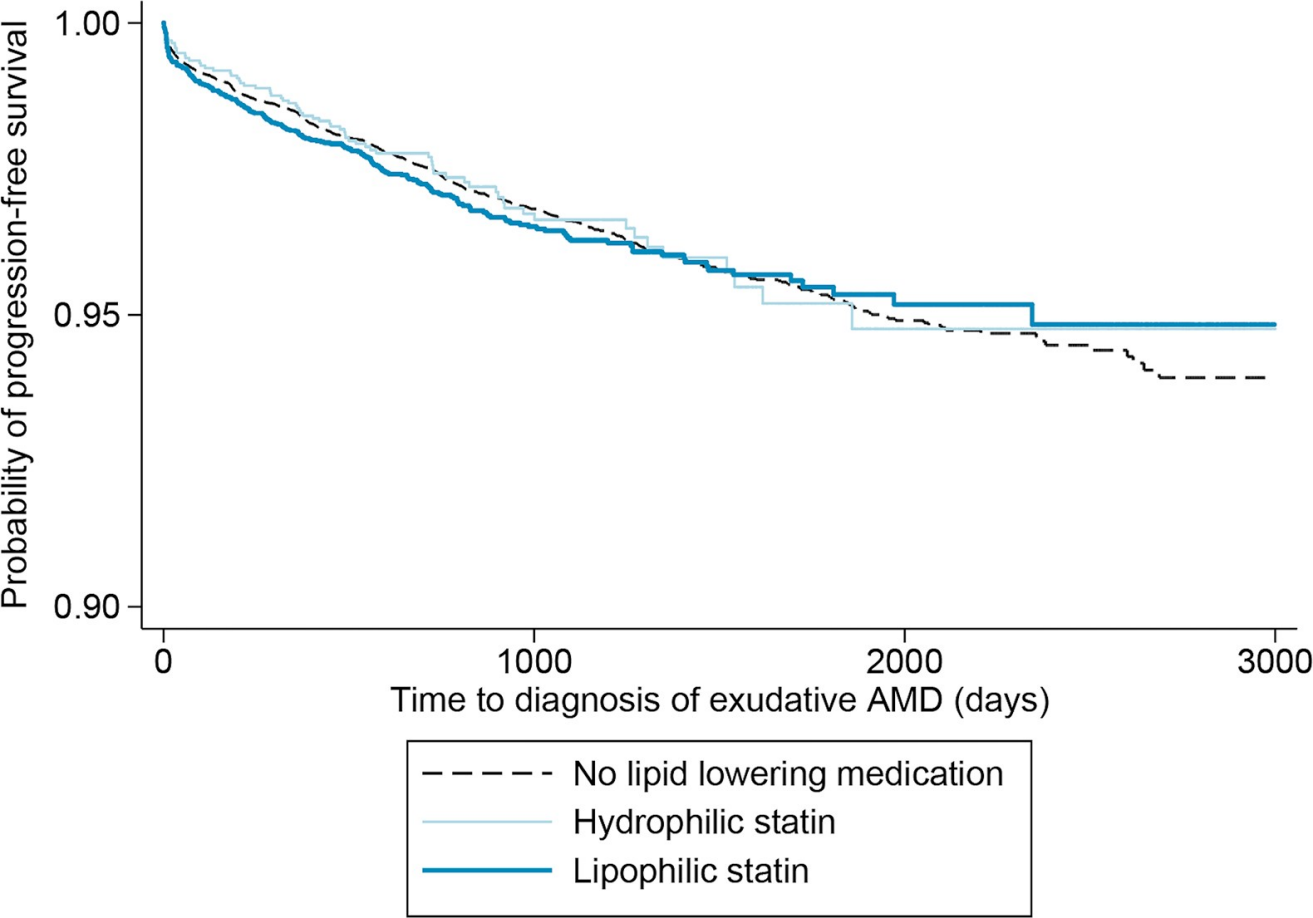
Time to progression from non-exudative to exudative age-related macular degeneration (AMD) by class of statin.

Unadjusted Cox models demonstrated a non-significantly increased risk of progression to exudative AMD and of anti-VEGF injection among patients on lipid-lowering medications, as well as among all subgroups with the exception of patients on fibrates and hydrophilic statins (**S2 Fig**). Similar outcomes were found with models that adjusted for patient attributes using matching based on propensity scores (**S3 Fig**). Unadjusted Kaplan-Meier curves of the progression to exudative AMD from time of index non-exudative AMD diagnosis, stratified by statin dose (**Fig 2**) and statin properties (**Fig 3**), illustrate the findings discussed above, with no progression to non-exudative AMD among patients on very-high-dose lipophilic statins.

All of these patients had a very-high-dose statin prescription that was unchanged within a year of their index non-exudative AMD diagnosis. Average follow-up time was 2.3 years (SD 1.0 years). In these unadjusted Kaplan-Meier curves there was no significant difference in progression to exudative AMD for patients taking high-dose lipophilic statins (simvastatin 40 mg to simvastatin 80 mg or equivalent) or low-dose lipophilic statins (statin dose lower than simvastatin 40mg or equivalent).

Average follow-up time for patients using hydrophilic and lipophilic statins was 2.8 years (SD 1.5 years) and 2.9 years (SD 1.6 years) respectively. In these unadjusted Kaplan-Meier curves there was no significant difference in progression to exudative AMD for patients taking lipophilic or hydrophilic statins compared to no lipid-lowering medications.

## Discussion

This is the largest observational study to specifically examine the effect of lipophilic statin dosage on progression to exudative AMD. In accordance with the findings of Vavvas et al. we found that no patients taking very-high-dose lipophilic statins converted from non-exudative to exudative AMD despite an expected incidence of 1 to 2 patients, though this finding was not statistically significant [23]. We found no significant relationship between exposure to high-dose or low-dose lipophilic statins and progression to exudative AMD, nor did we find a difference in AMD progression between lipophilic and hydrophilic statins despite their relative pharmacokinetic differences [25].

Prior studies on the relationship between statins and AMD have yielded heterogeneous results. Hall et al. were the first to propose a beneficial ophthalmic effect of statins in 2001 in a cross-sectional study of 660 patients, in which they found a 0.14 OR of AMD among the 379 statins users [18]. Similarly, a lower rate of progression of early AMD among statin users compared to non-statin users (3.6% vs. 13.0%) was subsequently reported in a study of 4,345 patients, though the effect was not statistically significant [20]. A nested case-control single-site study found that early AMD cases (n = 550) were 70% less likely to have received and filled a statin prescription than the 5,500 matched controls [29]. Two recent prospective clinical trials demonstrated a beneficial effect of lipophilic statins in non-exudative AMD [17, 23]; a prospective, double-masked trial of 114 patients randomized to simvastatin 40 mg (equivalent to our high-dose lipophilic statin group) versus placebo for three years found a significantly lower rate of AMD progression (54%) in the simvastatin group compared to the placebo group (70%), with a greater effect seen in the intermediate AMD group and those with the CFH genotype (Y402H) and no effect found in advanced AMD patients. A subsequent open-label prospective controlled trial of 24 AMD patients on atorvastatin 80 mg daily (equivalent to our very-high-dose lipophilic statin group) for one year found regression of large soft drusen and PEDs among 43.5% of patients at 1.5 year follow-up, with significant improvement in visual acuity among treatment responders compared to non-responders [23].

Despite these aforementioned studies suggesting a benefit of statins in AMD, we found no significant effect of statins on the progression of AMD, a finding supported by several prior population-based cohort and case-control studies [19, 21, 30–32]. The largest of these prior studies was a national claims database analysis of 10,753 patients with non-exudative AMD, in which a higher risk of progression from non-exudative to exudative AMD was found in those who used statins longer, though a lower risk of progression in those with normalized LDL and higher risk of progression in those with high HDL. A retrospective cohort study and a large prospective cohort study within AREDS2 similarly found no effect of statins on advanced AMD and on progression to late AMD, respectively [15, 33]. Systematic, non-systematic, and narrative reviews have all found insufficient evidence to conclude that statins have a role in preventing or delaying progression of AMD [6, 16, 34–36].

Strengths of our study include analysis of lipophilic statin exposure stratified by dosage, a distinction that has not been made in previous observational studies, and its strict control for confounders available within the database. Limitations include those inherent to database studies including the use of ICD coding, which is performed by physicians for billing purposes and may not accurately capture all pathology. More specifically, this study is limited by the inclusion of ICD-9 coding, which does not discriminate between stages of non-exudative or stages of exudative AMD and is therefore unable to capture potential variations in the statins’ effects in early and advanced AMD [34]. Differentiation of stages will be critical to determine timing of a possible primary prevention. This study is additionally limited by the use of NDC coding, as a statin prescription does not necessarily imply statin use. Furthermore, the total number of patients with NDC coding for a statin that included dose was significantly lower than the total number of patients taking statins leading to the potential for ascertainment bias. Lastly, following strict inclusion and exclusion criteria, the final cohort of patients taking very high dose statins was small. A prospective controlled study is required to evaluate dose response while controlling more accurately for risk factors unavailable in this data set (race/ethnicity, lipid profile, use of non-prescription AREDS2 formulations, basal metabolic rate) that may lead to residual confounding. Long duration of follow up in a prospective trial would be critical to determine the efficacy of very-high-dose lipophilic statins in preventing progression.

With a predicted 288 million people who will be affected by AMD by 2040, it is critical that we develop new means by which to prevent and treat AMD [37]. Although not statistically significant, we found that patients taking very-high-dose lipophilic statins did not progress to exudative AMD nor require anti-VEGF therapy over an average follow-up of 2.3 years. These results add to the growing body of research supporting a possible role for very-high-dose lipophilic statin therapy in preventing progression of non-exudative AMD, warranting further study. Future studies will benefit from additional ICD-10 classifiers of AMD to distinguish progression from earlier to advanced forms of non-exudative and exudative AMD.

## Supporting information

S1 Fig Exclusion criteria. Graphic representation of exclusion criteria applied to build final analytic sample.
(TIFF)

S2 Fig Forest plot of unadjusted cox proportional hazards models. Forest plot demonstrating unadjusted cox proportional hazards models of patients exposed to lipid-lowering medications and their progression to exudative AMD and/or need for treatment with anti-VEGF medication.
(TIFF)

S3 Fig Forest plot of adjusted cox proportional hazards models using propensity score matching. Forest plot demonstrating adjusted cox proportional hazards models using propensity score matching of patients exposed to lipid-lowering medications and their progression to exudative AMD and/or need for treatment with anti-VEGF medication.
(TIFF)
