# Supplementary figures and images for: Statins and the progression of age-related macular degeneration in the United States
Source: PLoS One. 2021 Aug 4;16(8):e0252878. doi: 10.1371/journal.pone.0252878 (PMC8336881; doi:10.1371/journal.pone.0252878)

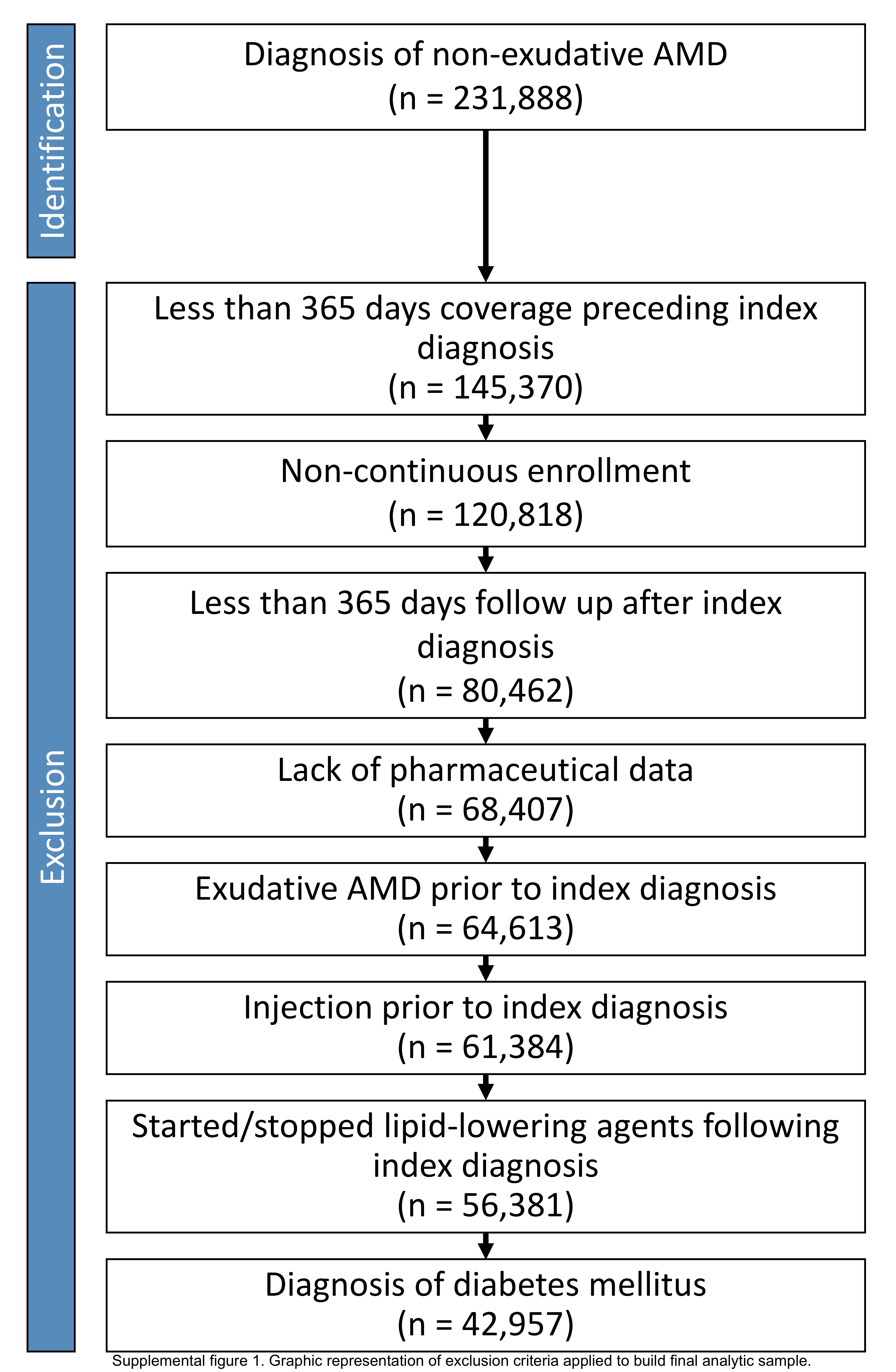

Supplement: S1 Fig — Graphic representation of exclusion criteria applied to build final analytic sample. (TIFF) [file pone.0252878.s001.tiff]

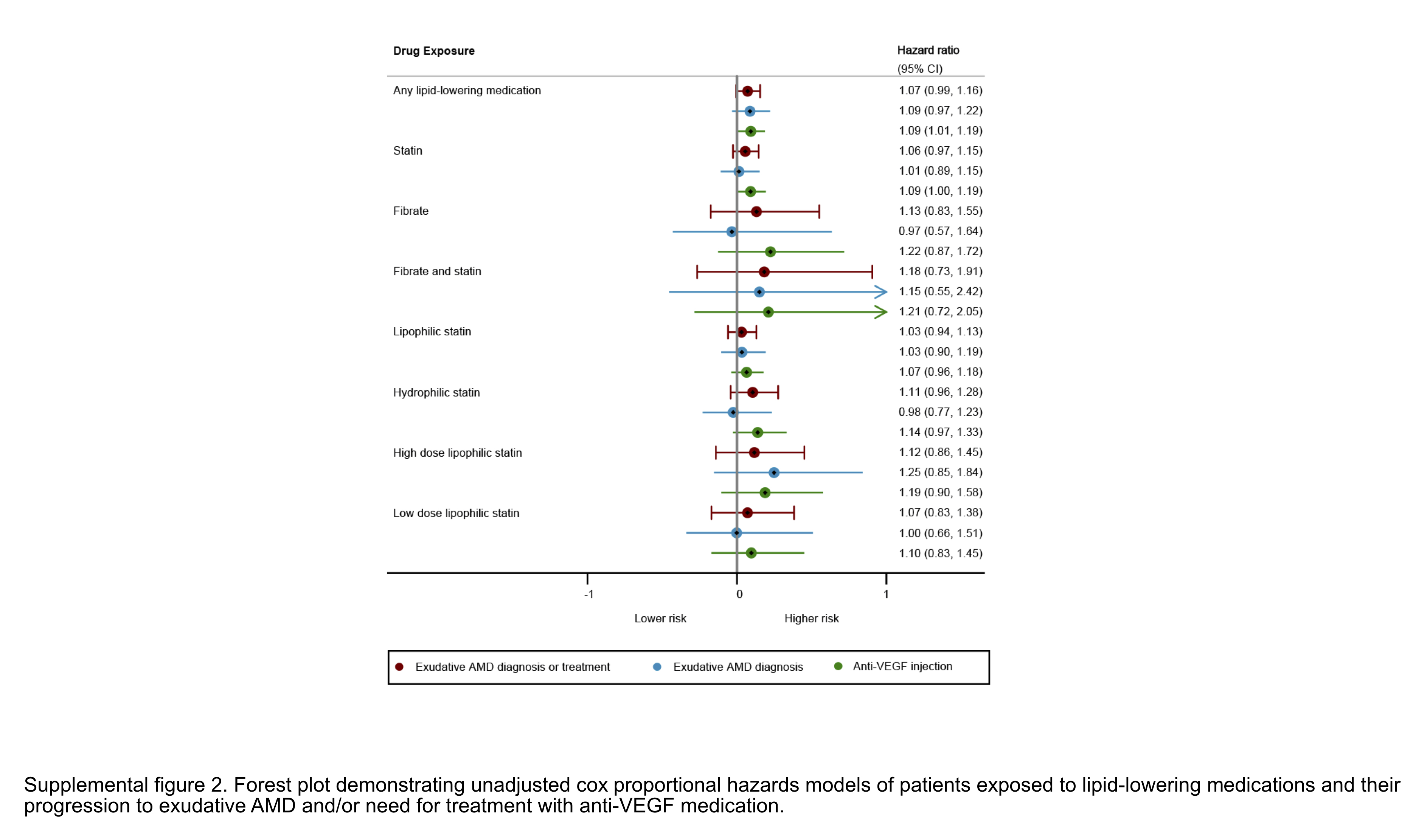

Supplement: S2 Fig — Forest plot demonstrating unadjusted cox proportional hazards models of patients exposed to lipid-lowering medications and their progression to exudative AMD and/or need for treatment with anti-VEGF medication. (TIFF) [file pone.0252878.s002.tiff]

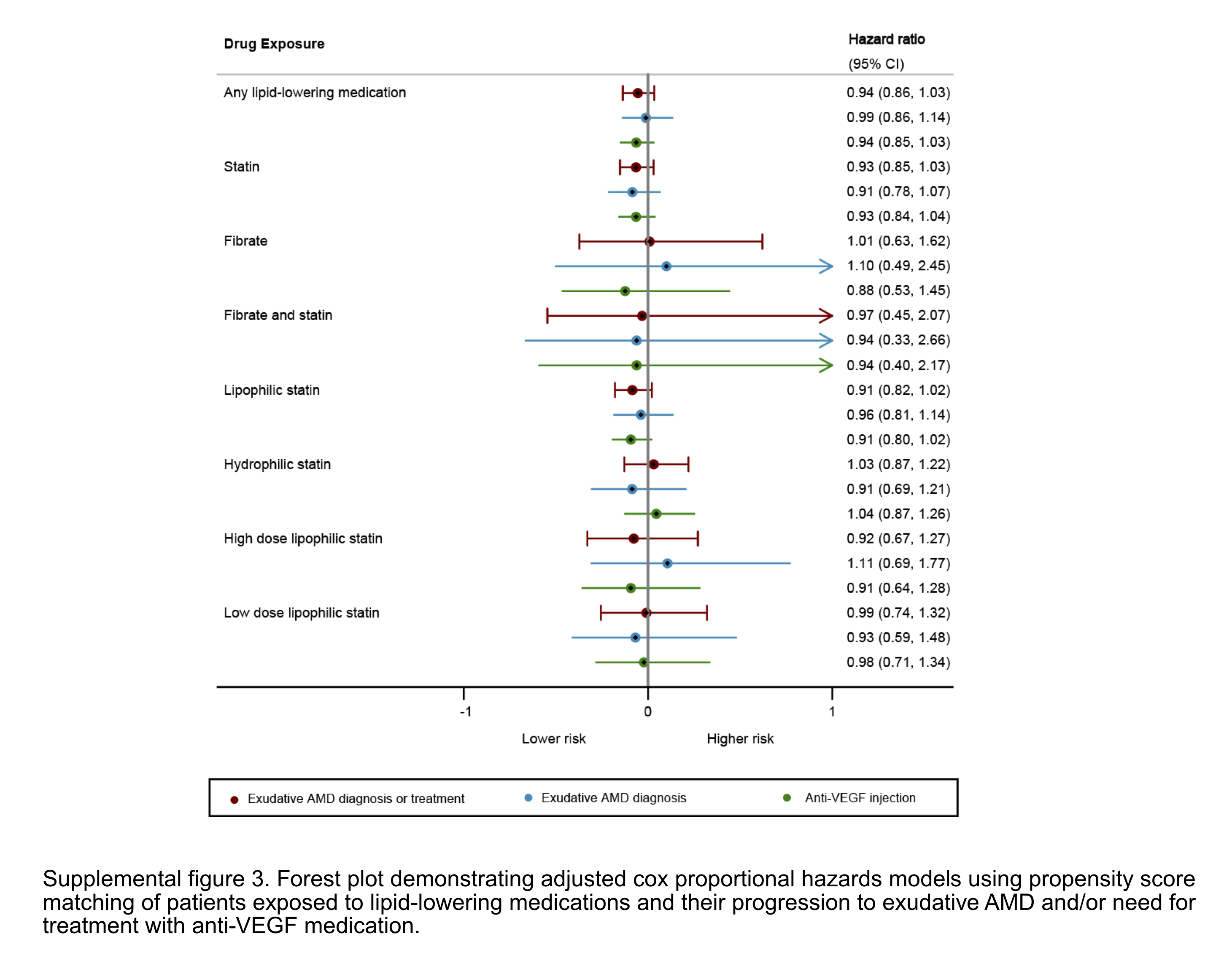

Supplement: S3 Fig — Forest plot demonstrating adjusted cox proportional hazards models using propensity score matching of patients exposed to lipid-lowering medications and their progression to exudative AMD and/or need for treatment with anti-VEGF medication. (TIFF) [file pone.0252878.s003.tiff]
